# Green Coconut Biorefinery: RSM and ANN–GA Optimization of Coconut Water Microfiltration with IntegratedTechno-Economic Analysis

**DOI:** 10.3390/foods15040623

**Published:** 2026-02-09

**Authors:** José Diogo da Rocha Viana, Moacir Jean Rodrigues, Arthur Claudio Rodrigues de Souza, Raimundo Marcelino da Silva Neto, Paulo Riceli Vasconcelos Ribeiro, José Carlos Cunha Petrus, Ana Paula Dionísio

**Affiliations:** 1 Programa de Pós-Graduação em Engenharia de Alimentos, Universidade Federal de Santa Catarina—UFSC, Florianópoli 88040-900, Brazil; diogo.rocha@posgrad.ufsc.br; 2 Programa de Pós-Graduação em Ciências Naturais, Universidade Estadual do Ceará—UECE, Fortaleza 60060-120, Brazil; 3 Embrapa Agroindústria Tropical—EMBRAPA, Fortaleza 60511-110, Brazil

**Keywords:** coconut water, artificial neural networks, response surface methodology, membrane separation process, fouling, economic modeling

## Abstract

The coconut water market continues to expand, but industrial supply is constrained by the high perishability of fresh coconut water and the need for stabilization routes that preserve quality. This study optimized crossflow microfiltration of coconut water using a silicon carbide (SiC) ceramic membrane, high permeability, chemical/thermal robustness, and cleanability, and assessed the techno-economic feasibility of a green coconut biorefinery producing microfiltered coconut water and coconut pulp. Pressure and temperature were modeled and optimized using a face-centered design (FCD) and artificial neural networks coupled with a genetic algorithm (ANN–GA), considering permeate flux and fouling index (*p* < 0.05). Both approaches converged to the same operating point, and experimental validation at 75 kPa and 30 °C achieved 605.32 ± 15.34 L h^−1^ m^−2^ and 82.79 ± 1.35% at VRR = 1. Sample-level fit statistics favored ANN (higher *R*^2^ and lower sample-level errors), whereas condition-wise grouped cross-validation (leave-one-condition-out) indicated higher predictivity and lower *RMSE_CV_* for the quadratic FCD/RSM models across experimental conditions, highlighting response-dependent generalization within the investigated domain. Fouling analysis indicated concentration polarization as the main resistance contribution and a flux-decline behavior best described by the intermediate blocking mechanism. A SuperPro Designer^®^ simulation over a 20-year project life indicated economic feasibility under baseline assumptions (Internal rate of return—IRR = 23.80%, Net present value—NPV = US$733,761, payback = 2.96 years), with profitability remaining attractive under ±10% selling-price variation. Overall, the process optimization and modeling outcomes align with the economic case, reinforcing the potential of this biorefinery concept for industrial deployment.

## 1. Introduction

Growing consumer interest in healthier beverage options has strengthened the position of natural fruit-based drinks, and coconut water has become one of the most recognized products in this segment, especially in tropical and subtropical regions [1,2,3]. FAOSTAT [4] reported a global coconut output of 62.41 million tons in 2022, with Indonesia, the Philippines, and India as the leading producers; Brazil placed fourth, reaching approximately 2.74 million tons (coconuts, in shell). In parallel to this agricultural base, ref. [5] highlights Brazil’s early role in shaping the international coconut water trade and its current status as a major industrial processor, supported by a supply chain focused on green coconuts for beverage production. Market data also reinforce the sector’s momentum: the global coconut water market was estimated at US$4.43 billion in 2024, while the Brazilian market generated roughly US$811.7 million in 2023, with strong growth expectations reported for both [6,7].

Despite this expansion, industrial production faces a central constraint: fresh coconut water is highly perishable, and conventional thermal stabilization can compromise sensory quality and key constituents. Therefore, non-thermal or mild-processing strategies that preserve “fresh-like” characteristics while ensuring microbiological stability are increasingly relevant [8]. In this context, tangential (crossflow) microfiltration has emerged as a promising route for clarifying fruit-based liquids while maintaining aroma and nutritional quality [9], with potential advantages in selectivity, clarification efficiency, and avoiding thermal damage compared with high-temperature processing [10,11]. In practical terms, microfiltration primarily removes suspended solids and colloidal material and can substantially reduce microbial load by retaining cells and associated particulates, which supports its frequent description as a “cold” clarification/stabilization step in beverage processing [12,13]. For coconut water specifically, enzymatic activity (e.g., oxidative enzymes associated with quality loss) is a recognized contributor to deterioration, and membrane-based clarification can help by limiting the particulate/colloidal fraction that carries or interacts with these components, while small dissolved constituents are largely expected to pass through microfiltration pores [14,15].

Microfiltration performance, however, depends on feed composition and operating conditions, including temperature and pressure, as well as matrix membrane interactions and, when applicable, enzymatic pretreatments [16]. In fruit-derived liquids, fouling is the main limitation to sustained flux, commonly associated with polysaccharides and macromolecular fractions such as pectin, cellulose, starch, and lignin, which promote concentration polarization and pore blocking, ultimately increasing hydraulic resistance [17]. Consequently, process optimization must balance productivity (permeate flux) and operational stability (fouling behavior), especially when the goal is industrial implementation.

Among the available membrane materials, polymeric modules are attractive from a cost standpoint, but their operation can be constrained by compaction and tighter limits on thermal/chemical sanitation, which can affect long-term stability under repeated CIP cycles. In contrast, ceramic membranes are widely adopted when robust cleaning and stable permeability are required, supporting higher operational resilience in clarification processes. In particular, silicon carbide (SiC) membranes have been highlighted for combining high water permeability with excellent chemical/corrosion resistance, enabling intensive cleaning/backwashing strategies and favoring longer service life in demanding separations [18,19].

The response surface methodology (RSM) is widely used to identify optimal processing conditions through designed experiments and empirical modeling of factor–response relationships [20,21]. In membrane-based clarification and fractionation of fruit-derived streams, recent studies have applied RSM to optimize operating variables (e.g., transmembrane pressure, temperature, and hydrodynamics) and quantify their interactions, including microfiltered banana juice [22], apple juice microfiltration with flux and fouling modeling [23], crossflow microfiltration of grape marc extract [24], and membrane-based concentration via osmotic distillation of red fruit juice [25]. However, membrane responses often exhibit strong non-linearity, limiting flux behavior, and coupled transport and fouling interactions, which motivate complementary data-driven approaches when responses depart from simple quadratic trends [26].

Artificial neural networks (ANNs) are well-suited to capture complex, non-linear relationships in membrane filtration, and they often improve predictive accuracy when flux and fouling responses show strong interactions and limiting flux regimes. This superiority over polynomial-type approaches has been reported for crossflow microfiltration and ultrafiltration, including studies comparing ANNs against traditional fouling/flux models [27,28,29,30]. More recent work and reviews further highlight the growing maturity of ANNs and broader AI tools for fouling prediction and performance forecasting in membrane processes [31,32,33].

Genetic algorithms (GAs) complement ANNs by enabling global optimization over the modeled domain through an evolutionary search for parameter combinations that maximize a chosen fitness function [34]. Recent studies have combined the RSM with an ANN–GA to simulate and optimize processing conditions in different food matrices, including cashew-based systems [35,36,37,38], sugarcane [39], lettuce [40], musambi [41], and pears [42]. However, in membrane-based processing of food liquids, integrated ANN–GA applications remain less common than conventional RSM, particularly in studies that also interpret fouling mechanisms and connect the optimized operating point to techno-economic performance [26,35].

In a green coconut biorefinery context, microfiltration optimization is not only relevant for process performance but also for economic feasibility, because flux stability and fouling directly influence capacity utilization and operating costs. Therefore, this study aimed to (i) optimize pressure and temperature in tangential microfiltration of coconut water using a silicon carbide (SiC) membrane, (ii) compare Face-Centered Design (FCD) and ANN–GA as predictive and optimization tools, (iii) characterize fouling behavior to support scale-up, and (iv) perform a techno-economic assessment of a conceptual biorefinery producing microfiltered coconut water and coconut pulp to verify whether the optimized conditions translate into an economically viable industrial scenario.

## 2. Materials and Methods

### 2.1. Obtaining Green Coconut Water (Cocos nucifera *L.*) and Experimental Diagram of the Study

The coconut water was supplied by Nosso Coco (Paraipaba Agroindustrial Ltda–Located in the municipality of Paraipaba, CE, Brazil.) already containing potassium metabisulfite—K_2_S_2_O_5_ (≈10 mg L^−1^, supplier specification)—as an antioxidant; no additional metabisulfite was introduced by the authors. This practice is widely used to mitigate oxidative browning and pink discoloration during the processing and storage of coconut water [43].

**Figure 1:**
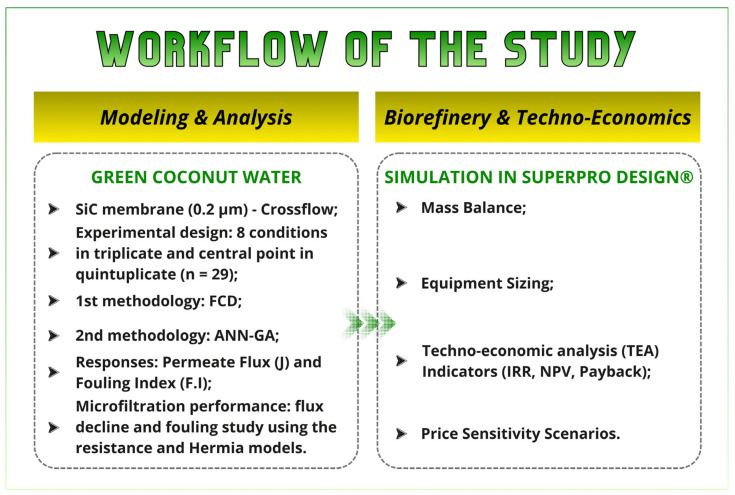
Overview of the research workflow and integration steps.

The schematic diagram of the optimization process up to an economic feasibility study of the green coconut biorefinery is described in Figure 1.

### 2.2. Experimental Design

The studies started with an experimental design aimed at optimizing the operational parameters (pressure—kPa; temperature—°C) of the microfiltration process; therefore, the variables were independent of the design. The design was a 2^2^ type with 5 central points, yielding 13 experiments per treatment. All experiments were performed using a single industrial batch of green coconut water supplied by the same manufacturer. Upon receipt, the batch was divided into 29 packages of 6.0 L each and kept in refrigerated storage before each experimental run to minimize stratification of the composition. For each FCD condition, one package was removed and processed as an independent microfiltration run. Therefore, the variability reported (Table 1) as mean ± standard deviation reflects the experimental replicates between series within a batch (not repeated analytical measurements of the same permeate sample). The design used a silicon carbide (SiC) membrane with an average pore diameter of 0.2 µm. The dependent variables in both designs were permeate flux (L h^−1^ m^−2^) and fouling index (%).

Given the above, it was determined that a face-centered design (FCD) would be the most appropriate approach to optimize the pressure and temperature parameters because such planning focuses on optimization within the ranges established by such methodology (i.e., the minimum (−1), the central point (0), and the maximum (+1) coded values)—Figure S1; Supplementary Material. Table 1 presents the levels of the design that were performed.

#### 2.2.1. Response Surface Methodology—RSM

The face-centered design (FCD) follows a mathematical model that indicates the effect of variables in linear terms, quadratic terms, and interactions, relating the variables (*X_i_*, i = 1, 2) by a second degree polynomial given by Equation (1), where *Y* is the two experimental responses, *X_i_* and *X_j_* are the levels of variables, *β*_0_ is the constant term, *β_i_* is the coefficients of the linear terms, *β_ii_* is the coefficients of the quadratic terms, and *β_ij_* is the coefficients of the interactions terms.

The Protimiza Experimental Design software program [44] was used for the FCD of the experimental planning performed, and analysis of variance (ANOVA) was used at a significance level of *p* < 0.05.

(1) $$Y=\beta_{0}+\sum_{i=1}^{j}\beta_{i}X_{j}+\sum_{i=1}^{j}\beta_{ii}X_{i}^{2}+\sum_{i\neq j=1}^{j}\beta_{ij}X_{i}X_{j}$$

#### 2.2.2. Artificial Neural Networks (ANNs) Associated with Genetic Algorithms (GAs)

**Figure 2:**
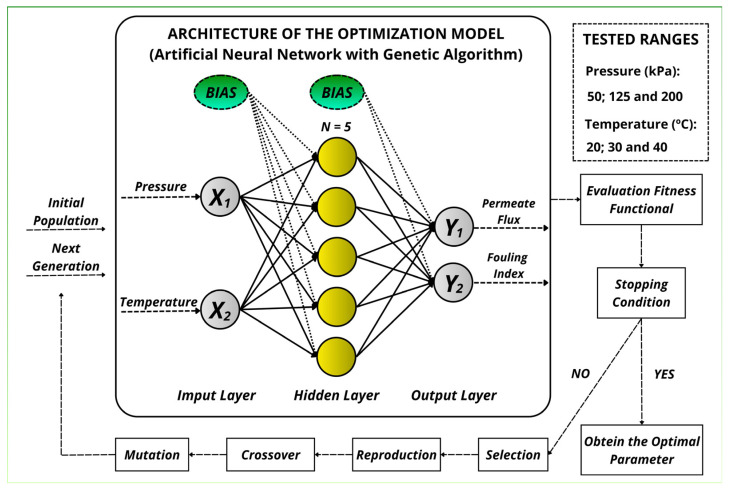
Schematic diagram of the ANN and genetic algorithm (GA) models developed for the experimental design.

ANNs and a genetic algorithm (GA) were employed for modeling and optimization of process parameters using the neural adaptation application and optimization tool, respectively, of the MATLAB^®^ software program (Version R2024b, The MathWorks, Inc., Natick, MA, USA). A multilayer feed-forward (MFF) network, output neurons, and hidden sigmoid neurons were used in the ANN. The multilayer feed-forward comprises a chain of layers, and the initial layer is connected to the input of the network. Figure 2 illustrates the optimization scheme of the ANN and the genetic algorithm used for the design, as implemented by the response surface methodology.

The neurons in each successive layer contain a bias value and are connected to the neurons in the previous layer, while the final layer is responsible for the net’s output.

The selected MFF had an input layer, one hidden layer, and an output layer. A single hidden layer was adopted to keep the model compact and reduce the risk of overfitting within the limited experimental domain [45].

The input layer contained two neurons [pressure (X_1_) and temperature (X_2_)], and the output layer contained two neurons [permeate flux (Y_1_) and fouling index (Y_2_)]. The network was trained using the Levenberg–Marquardt backpropagation algorithm (*trainlm*) [46]. The dataset was randomly divided into training, validation, and testing subsets (70%, 15%, and 15%, respectively) for training diagnostics. Although the Face-Centered Design comprises 13 unique pressure–temperature conditions, the ANN was trained using the full experimental dataset, including replicates (*n* = 29 samples: five replicates at the center point and three replicates at each of the remaining eight points). Therefore, the 70/15/15 split was applied at the sample level (not at the condition level), resulting in 20/4/5 samples for training/validation/test in a typical random split. To reduce overfitting, a compact ANN architecture was used (a single hidden layer with 5 neurons), and L2 (ℓ2) regularization was applied during training. Regularization was implemented in MATLAB^®^ through the performance-function regularization setting (regularization parameter = 0.10). Model selection was performed through multiple random initializations, retaining the network with the best validation performance under identical preprocessing and training settings. The hidden layer used a hyperbolic tangent sigmoid (*tansig*) transfer function, and the output layer used a linear (purelin) transfer function. Because *tansig* is bounded between −1 and +1, input–output data were normalized to this range prior to training using mapminmax, and then converted back to real units after the output layer [47]. The transfer functions are given in Equations (2) and (3). No transfer function was used for the input layer.

(2) tansig n=1[1+e−2n]−1

(3) $$purelin\;\left(n\right)=n$$

In addition to the internal random split used for training diagnostics, model predictivity within the investigated domain was assessed using grouped cross-validation (leave-one-condition-out). In each fold, all replicates from one pressure–temperature condition were held out for testing, while models were trained on the remaining conditions and their replicates. This strategy prevents replicate leakage across folds and provides a stricter estimate of predictive performance for both the ANN and the quadratic RSM model.

### 2.3. Crossflow Microfiltration Setups (CFMF)

The studies were performed with three crossflow microfiltration setups: silicon carbide membranes (SiC—Crystar™ FT250). Figure S2 (Supplementary Material) details the crossflow microfiltration setups and their membrane specifications (SiC; average pore diameter 0.2 µm). Operating conditions were harmonized across setups by matching transmembrane pressure, temperature, and crossflow velocity; all results are reported per unit membrane area (L h^−1^ m^−2^). Clean-in-place protocols restored ≥95% of the initial clean-water flux for all membranes, ensuring fair comparability.

All trials were run on the same pilot skid equipped with a 10 L feed tank and a NEMO 1.0 CV helical rotor positive displacement pump. Pump speed was adjusted to deliver an average pipe velocity of 6 m s^−1^. The system dead volume was ≈1.0 L (pump + lines + module). Crossflow microfiltration was conducted at a transmembrane pressure (ΔP_TM_) of 75 kPa (0.75 bar) and a bulk temperature of 30 °C.

#### 2.3.1. Model Calculations of the Tangential Microfiltration Process

The permeate flux was calculated every 2 min of the process. The permeate flux (*J_p_*—L h^−1^ m^−2^), volumetric retention ratio (*VRR*), transmission (*T*), and retention/rejection (*R*) coefficients were calculated according to Equations (4)–(7).

(4) JP=VPt×Am

(5) VRR=VfVr

(6) T=CPCA×100

(7) R=1−CPCA×100

Regarding the permeate flux (*J_p_*), *V_P_* corresponds to the permeate volume collected in liters, *A_M_ is* the membrane area in m^2^, and *t* is the time interval at which the permeate was collected in seconds. Regarding the volumetric retention ratio (*VRR*), *V_F_* and *V_R_* correspond to the total feed volume and the retentate volume, respectively. For the transmission and rejection coefficients, “*C_P_*” and “*C_A_*” denote the solute concentrations in the permeate and feed, respectively (mg L^−1^).

#### 2.3.2. Study of Resistance and Fouling Phenomena

The resistances and permeances of the microfiltration process were calculated according to the literature using Equations (8)–(18), as described in Table 2.

As a complementary interpretive tool, we adopt the critical-flux notion—the flux below which fouling is not observed for a given feed and hydrodynamics—and use it qualitatively to relate resistance partitions to operating conditions [51,52].

The models proposed by Hermia [53] were used to identify the predominant fouling mechanism in the evaluated processes. These models incorporate four fouling mechanisms (complete pore blockage, internal pore blockage, partial pore blockage, and cake/torta formation). These models are described in Table 3.

### 2.4. Clean in Place (CIP) Protocols

Each membrane type was cleaned according to a protocol optimized in preliminary screening tests and in accordance with manufacturer recommendations, ensuring full restoration of initial permeability without damaging the membrane. All protocols began with a warm-water pre-rinse (30 °C, 10 min) to remove loosely bound deposits, followed by an alkaline wash and an acidic rinse, then a final water rinse to neutrality.

Specifically, SiC membranes were subjected to a total-recirculation cycle of an alkaline solution (3.0% *w*/*v* NaOH + 1.0% *w*/*v* NaClO) for 10 min at 80 °C, followed by an alkaline backwash with the same solution for another 10 min at 80 °C, rinsing to pH 7.0, a further total-recirculation cycle of 1.0% *w*/*v* HNO_3_ for 20 min, and a final rinse to pH 7.0. All reagents were analytical grade, and rinses were performed with deionized water (conductivity < 10 µS).

Cleaning efficacy was quantified by the clean-water flux recovery ratio (post-CIP clean-water flux relative to the initial clean-water flux). Recovery ratios above 95% indicate that irreversible fouling after CIP was minimal, and that most of the performance loss observed during coconut-water filtration was reversible (e.g., concentration polarization). By tailoring the chemistry and temperature to each material yet evaluating flux recovery on a normalized basis, we ensured that all membranes could be benchmarked fairly under identical operating conditions.

### 2.5. Technical and Economic Analysis

SuperPro Designer^®^ Version 11 software (Intelligen, Inc., Scotch Plains, NJ, USA) was used to perform the mass and energy balance of the conceptual green coconut biorefinery (GCB) and thus perform its technical and economic analysis. The year 2023 was considered the base year for the project. The lifetime was 20 years, with a construction period of 30 months and a start-up time of 4 months. The US dollar (USD) was considered the currency, and the exchange rate of US$1 = R$5.00 (Brazilian real) was used, which was the average value in 2023 [56]. For the financial analysis, an inflation rate of 4% and a discount rate of 13% per annum were adopted, based on the average Brazilian basic interest rate (SELIC) [57]. This discount rate represents the Minimum Acceptable Rate of Return (MARR) used to calculate the Net Present Value (NPV) of the project.

The equipment purchase cost (EPC) was estimated based on equipment sizing after mass and energy balances were performed in SuperPro Designer. For equipment whose prices were not in the software database, the cost-scaling method was applied using Equation (27).

(27) EPC=C0QQ0a

where “*Q*” is the desired capacity of the new equipment; *C*_0_ is the cost of the reference equipment at capacity *Q*_0_; and the exponent ‘a’ represents the scale factor (adopted as 0.6), which quantifies the relationship between cost and the increase in productive capacity [58].

The industrial plant was designed to process 1 ton of green coconut per batch, with 300 days of operation per year. A base salary was considered, according to labor agreements registered with the Brazilian Ministry of Labor (MTE), of $1.86 h^−1^ (Registration number CE000046/2024) for operators and $2.60 h^−1^ (Registration number SP000032/2024) for supervisors [59]. The composition of the green coconut used was as follows: husk (33.6%), shell (16.5%), albumen (30.4%), and coconut water (19.5%) [60].

Based on the information provided and that contained in its database, SuperPro Designer (SPD) calculated the equipment acquisition costs and plant construction costs. Two sources of revenue were considered: microfiltered coconut water ($1.68 L^−1^) and pulp ($2.80 kg^−1^). The prices used here were simulated as factory prices, i.e., lower (30% less) than the prices of products already marketed in Brazil, which were $2.40 L^−1^ for coconut water and $4.00 kg^−1^ for pulp [61,62]. In the SPD, a box containing 10 units of 1 L of coconut water and 10 units of 0.4 kg of pulp was considered a production unit. Thus, the prices were $16.80 per box of coconut water and $11.2 per box of pulp. Based on these prices, a sensitivity analysis was performed with variations of ±10% and ±20%. The effect of these variations on the Internal Rate of Return (IRR), Net Present Value (NPV), and Payback time was analyzed.

### 2.6. Mineral Analysis

The samples were digested following the procedures of previous studies [63,64]. A sample (1.0 g) was placed in a digestion tube and reacted for 12 h with nitric acid and perchloric acid (3:1, *v*/*v*). The digestion was carried out using a dry block digester at 250 °C for 4 h. After cooling, the volume was brought up to 50 mL with deionized water and filtered through a quantitative filter paper. The samples were analyzed by inductively coupled plasma optical emission spectrometry (ICP/OES) (Agilent, model 5100, Mulgrave, Australia) for phosphorus (P), potassium (K), calcium (Ca), magnesium (Mg), sodium (Na), manganese (Mn), Iron (Fe), sulfur (S), zinc (Zn), and copper (Cu).

### 2.7. Statistical Analysis

The performances of the models developed using FCD and ANN were compared using statistical parameters, including mean absolute deviation (*AAD*), mean squared error (*MSE*), normalized mean squared error (*NMSE*), root mean squared error (*RMSE*), normalized root mean squared error (*NRMSE*), mean percentage error (*MPE*), and coefficient of determination (*R*^2^), calculated using the formulas given in Equations (28)–(34). In addition, model generalizability was assessed by condition-wise grouped cross-validation (leave-one-condition-out), reporting the cross-validated predictive coefficient (*Q*^2^) and the cross-validated root mean squared error (*RMSE_CV_*) (Equations (35) and (36)). The model showing lower *AAD*, *MSE*/*NMSE*, *RMSE*/*NRMSE*, *MPE*, and *RMSE_CV_*, together with higher *R*^2^ and *Q*^2^, was considered more accurate and more predictive within the investigated domain; when *Q*^2^ was negative, this was interpreted as poorer condition-wise predictivity than using the overall experimental mean as a baseline predictor.

(28) $$AAD=\frac{\sum \left|X_{P}-X_{A}\right|}{n}$$

(29) $$MSE=\frac{\sum {\left(X_{P}-X_{A}\right)}^{2}}{n}$$

(30) $$NMSE=\frac{MSE}{X_{M}}$$

(31) $$MPE=\frac{100}{n}\sum \left|\frac{X_{P}-X_{A}}{X_{P}}\right|$$

(32) $$RSME=\sqrt{MSE}$$

(33) $$NRSME=\frac{RSME}{X_{M}}$$

(34) $$R^{2}=1-\frac{\sum (X_{P}-X_{A}{)}^{2}}{\sum (X_{P}-X_{M}{)}^{2}}$$

(35) $$Q^{2}=1-\frac{\sum (X_{A}-X_{P}^{CV}{)}^{2}}{\sum (X_{P}-X_{M}{)}^{2}}$$

(36) $${RMSE}_{CV}=\sqrt{\frac{1}{n}}\sum (X_{A}-X_{P}^{CV})$$

where “*X_P_*” is the predicted data, “*X_A_*” is the experimental data, “*X_M_*” is the average experimental data, “$X_{P}^{CV}$” is the model’s prediction when its condition (pressure-temperature) was left out of the adjustment, and “*n*” is the number of experiments performed.

## 3. Results and Discussion

### 3.1. Analysis of the Effects of Pressure and Temperature on Experimental Design

According to Table 1 (Section 2.2), it can be observed that pressure had a negative effect, meaning it is harmful to the dependent variables, as can be seen by comparing experiments 1–2 and 3–4 in Table 2, where the permeate flow does not change with increasing pressure, but the fouling index is significantly higher in experiments where there is an increase in pressure. The opposite behavior is observed when evaluating the effect of temperature, as confirmed by comparing experiments 1–3 and 2–4, where pressure is maintained but temperature increases, leading to higher flows and no significant change in fouling.

We can confirm that the temperature increase has a bigger influence when compared to a pressure increase in the experiments referring to the central point (9–13), where the flows were the highest (656.11 ± 15.25) and the fouling index presented the lowest values (85.19 ± 1.06), confirming a strong indication that parameters closer to the central point are the best for the evaluated responses.

Therefore, there is a tendency for the best parameters to be close to the central point. This can be confirmed by the evaluation of the effects and the response surface and contour plots generated by the applied response surface methodology. This can be confirmed by the information shown in Table 4 and Figure 2.

Considering the balance between driving force (pressure) and boundary-layer effects (concentration polarization and fouling), the trends observed in Table 1 and Figure 2 are consistent with what is expected for crossflow microfiltration of coconut water and other low-acid beverages. Raising the operating temperature typically lowers viscosity and can increase mass-transfer coefficients, which favors a higher permeate flux at the same transmembrane pressure [15]. However, the increase is not always linear, as concentration polarization and deposit-layer formation can quickly impose additional hydraulic resistance, producing a flux plateau even when the pressure is increased [65]. Studies on the membrane processing of coconut water report the same qualitative behavior: flux increases with pressure and temperature up to a limiting regime governed by polarization and fouling [66]. A set of operating conditions is selected to balance throughput and operational stability [26].

In practical terms, this indicates that the most suitable operating region is not necessarily the one at the highest pressure, but rather a window that avoids entering the limiting regime and helps maintain a stable permeate flux over time [52,67]. The implications of this behavior for irreversible fouling, resistance buildup, and cleaning frequency are explained in detail in Section 3.2.

#### 3.1.1. Statistical Analysis of the Experimental Design—RSM

Table S1 (Supplementary Material) expresses the regression coefficient values for the dependent variables.

The parameters were studied and considered significant at a significance level of *p* < 0.05, so only the linear term (L) of the pressure was not considered significant in the permeate flow response. The linear term (L) of temperature and the quadratic term (Q) of pressure were not considered significant in the fouling response.

Next, *F_cal_ > F_tab_* and the correlation coefficients (*R*^2^) are greater than 0.90 for both dependent variables in Table S2 (Supplementary Material), so it can be stated that the proposed models (Equations (1) and (2)—Table S2) fit the experimental data well.

Face-centered designs (FCDs) are frequently selected in response-surface studies when a full three-level exploration is desired without the star points extending beyond the factorial space. In FCD, the axial points lie on the faces of the cube (α = 1), which simplifies experimentation and avoids factor levels that may be impractical or unsafe for food processes, while still enabling estimation of curvature and interaction terms in second-order models. Therefore, the adequacy of Equations (32) and (33), supported by the high coefficients of determination and the *F_test_* criteria reported in Table 4, indicates that the selected design space and polynomial structure are appropriate for describing the permeate-flux and fouling-index responses within the studied domain [68,69].

**Figure 3:**
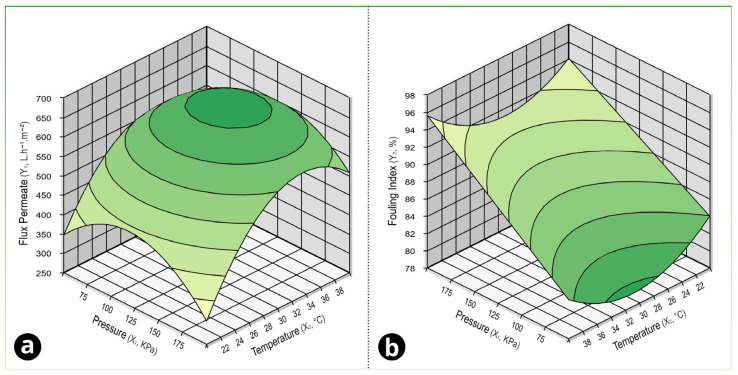
Response surfaces of the experimental design for the dependent variables: (**a**) permeate flux rate and (**b**) fouling index.

According to Figure 3, we observe the response surfaces for the independent variables and their respective optimal zones.

The optimal parameters for the permeate flow response would be a pressure of 128.5 kPa and a temperature of 32.90 °C, which would correspond to a flow rate of 671.84 ± 12.89 L h^−1^ m^−2^, whereas for the index fouling response, the best pressure would be 78.71 kPa and 31.10 °C, which would correspond to a fouling of 78.71 ± 0.51%.

Because the study targets two responses with different operational priorities, optimization should be interpreted as a trade-off rather than a single-point maximum. Higher flux directly increases hourly productivity, but it can also increase the rate of deposit buildup and accelerate the transition from pore blocking to cake/gel-layer control. Conversely, conditions that minimize the fouling index may sacrifice some throughput but support longer continuous operation, which is often the dominant lever for industrial performance when considering cleaning and downtime. This multi-response perspective aligns with the membrane filtration literature, where process windows are commonly defined by sustainable flux and fouling propensity, rather than only by the initial flux value [26,52,67].

#### 3.1.2. Statistical Analysis of the Experimental Design—ANN

The ANN was trained using the full experimental dataset, including replicates, whereas the internal random split into training/validation/test subsets was applied only for training diagnostics (performance curve, regression plots, and residual distributions), following common practice in ANN modeling of experimental processes [70]. A compact topology (a single hidden layer) with L2 (ℓ2) regularization and multiple random initializations was used, as described in Materials and Methods.

The training–performance trend and residual distributions are summarized in Figure S3 (Supplementary Material): the error histograms (target−output) for the training/validation/test subsets (Figure S3a,b) are centered close to zero, indicating no strong systematic bias in the fitted model. Following common ANN validation practice for small experimental datasets, regression diagnostics are reported in Figure S3c–f (training, validation, test, and all samples) and were generated using pooled target–output pairs across both ANN outputs (permeate flux and fouling index). The plots show slopes close to unity and high coefficients of determination (*R*^2^ ≈ 0.99), indicating close agreement between predicted and experimental values within the sampled pressure–temperature domain. In addition to these sample-level diagnostics, model generalizability across experimental conditions was evaluated using grouped cross-validation (leave-one-condition-out), and the corresponding cross-validated indicators are reported separately alongside the cross-validation metrics.

**Figure 4:**
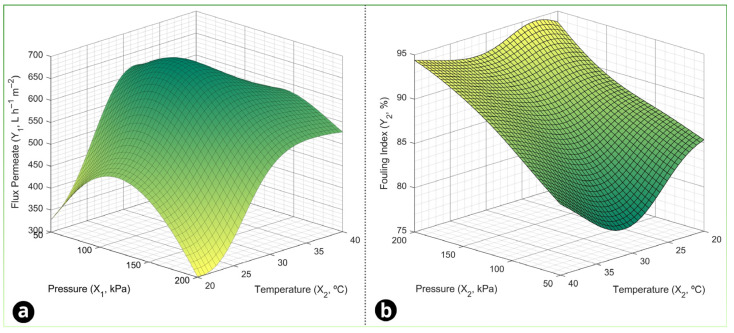
Response surfaces of the experimental design for the dependent variables (**a**) permeate flux rate and (**b**) fouling index in the artificial neural network (ANN) methodology.

According to Figure 4, we can observe that there is a convergence between the responses used, as there is a pressure–temperature overlap region where the models simultaneously indicate higher *J_p_* and lower F.I, as in the FCD methodology, which allows us to identify and choose an optimal point to finally obtain an optimization of the parameters proposed by the experimental design. The increase in pressure raised the *J_p_*, while the F.I tended to increase at higher pressure levels due to the accumulation of deposits. The increase in temperature favored the *J_p_* by reducing viscosity and can mitigate the F.I in the membrane, improving hydrodynamics and mass transfer. Thus, the ideal set point was chosen in the region where these trends overlap.

The optimal parameters for the permeate flux response in the neural network methodology would be a pressure of 103 kPa and a temperature of 30 °C, which would correspond to a flow of 676 L h^−1^ m^−2^, while the best pressure for the fouling index response would be 50 kPa and 31 °C, which would correspond to a fouling of 78%.

The use of artificial neural networks (ANNs) in this work is particularly valuable because membrane filtration responses are frequently nonlinear and may involve complex interactions that are not fully captured by second-order polynomials, especially when fouling dynamics and temperature-dependent physicochemical effects are present. ANNs learn the input–output mapping directly from the data and can approximate highly nonlinear relationships without requiring an explicit mechanistic form, which makes them well-suited statistical surrogate models for process optimization. Recent reviews on AI for membrane processes highlight that data-driven models, including ANN architectures, often provide higher predictive accuracy than classical regressions for fouling-related variables, particularly when multiple operating factors interact [32]. In food engineering, ANN-based optimization is still less common than RSM, but it has been increasingly used to model and optimize unit operations where nonlinearity is pronounced; hybrid ANN-GA approaches are widely used to search for global optima when the response surface is highly nonlinear and gradient information is not available [32,71].

In the specific context of membrane operations, published applications remain relatively limited, which reinforces the methodological contribution of using ANN as a complementary route to statistical modeling, together with the experimental design framework adopted here. In membrane science, ANN models can be widely used as empirical substitutes for flux decline and fouling, as they capture nonlinear interactions without imposing a predefined functional form [28,29,31]. In beverage filtration, ANN has also been used to model microfiltration performance and can outperform polynomial models when responses exhibit strong curvature [72].

#### 3.1.3. Comparison Between Statistical Methodologies (FCD–ANN)

Table 4 summarizes the predictive performance of the FCD (quadratic RSM) and ANN models for permeate flux (*J_p_*) and fouling index (F.I) using complementary goodness-of-fit and error metrics (*R*^2^, *AAD*, *MSE*, *RMSE*, *MPE*, *NMSE*, and *NRMSE*) computed at the sample level (i.e., using all individual observations, including replicates), and it also reports condition-wise cross-validation results using *Q*^2^ and *RMSE_CV_* obtained by leave-one-condition-out validation, where all replicates from one pressure–temperature condition are held out together.

This multi-metric approach is consistent with common practice in data-driven modeling of membrane filtration, where *R*^2^ is interpreted alongside absolute and normalized error indices to provide a more complete picture of model performance rather than relying on a single statistic [31]. Using multiple indicators is important because *R*^2^ alone does not fully describe predictive accuracy, whereas error-based indices quantify the magnitude of deviations (e.g., *RMSE*), normalize errors to the response scale (*NRMSE*), and help identify systematic bias (*MPE*) when comparing alternative models for the same process [73].

At the sample level, the ANN produced higher *R*^2^ values (closer to 1.0) and lower errors than the corresponding FCD models for both *J_p_* and F.I., which agrees with literature showing that ANN can outperform polynomial-type models when membrane responses depart from simple quadratic trends and reflect coupled transport–fouling interactions [28,29]. However, Table 4 also highlights that predictive performance can change under stricter generalization testing at the condition level: *Q*^2^ and *RMSE_CV_* reflect the ability to predict unseen pressure–temperature conditions without replicate leakage. In this context, a negative *Q*^2^ (observed for ANN in *J_p_*) indicates that, under the leave-one-condition-out scheme, the model predictions were, on average, less accurate than using the overall experimental mean as a baseline predictor; this does not contradict the high sample-level *R*^2^, but rather indicates that the ANN fit can be sensitive to limited coverage of the design space when evaluated on fully held-out operating conditions. Importantly, despite these differences in condition-wise predictivity, both modeling approaches converged to the same optimal operating region, which strengthens confidence in the selected pressure–temperature setpoint within the investigated domain.

#### 3.1.4. Genetic Algorithm (GA) Optimization and Experimental Validation

**Figure 5:**
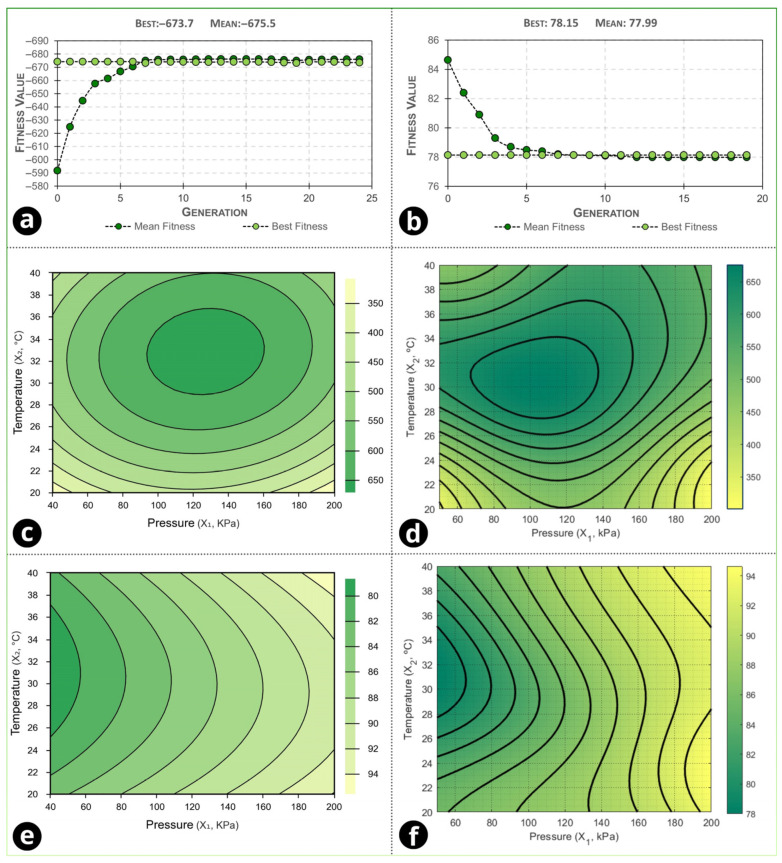
(**a**) Variation in the fitness value of the response of (**a**) permeate flux and (**b**) fouling index in relation to generations during genetic algorithm optimization and contour surfaces of the dependent variables: (**c**,**d**) permeate flux and (**e**,**f**) fouling index in RSM and ANN methodologies.

Genetic algorithm (GA) optimization was used to identify pressure–temperature setpoints within the experimental domain that either maximize permeate flux (*J_p_*) or minimize the fouling index (F.I) based on the ANN-predicted response surfaces. In MATLAB, pressure and temperature were defined as the decision variables, with bounds constrained to the minimum and maximum values tested in the face-centered design. The GA was implemented as a single-objective optimization run separately for each response (i.e., one GA run for *J_p_* and one GA run for FI), rather than as a multiobjective/Pareto procedure. The objective direction was implemented in the fitness formulation evaluated by the trained ANN: for *J_p_*, the fitness was set as fitness = −*J_p_* so that maximization could be solved using MATLAB’s minimization framework; for F.I, fitness = F.I. The solver was configured with a population size of 120, 50 generations, preservation of 5% of the population, an adaptive-feasible mutation, and a stall-generation limit of 8. After this user-defined setup, the optimization proceeded automatically, with selection, crossover, mutation, and population updates executed at each generation without manual intervention. Figure 5a,b shows the evolution of best and mean fitness across generations for each run, and the curves stabilized after the sixth generation. The figure shows that there was no change in the fitness value after the sixth generation for both responses, and the fitness value obtained was −673.70 and 78.15 for the permeate flux and fouling index responses, respectively. It should be noted that, for the permeate-flux optimization, the GA fitness was formulated as the negative of the predicted flux (fitness = −*J_p_*) so that a maximization problem could be solved with MATLAB’s default minimization framework; therefore, negative “best fitness” values (e.g., −673.70) reflect a higher predicted flux rather than a negative physical flux.

The GA extrema predicted by the ANN were 103.31 kPa and 30.39 °C, yielding *J_p_* = 675.66 L h^−1^ m^−2^ for the flux-maximization run, and 50.00 kPa and 31.21 °C, yielding FI = 77.99% for the fouling-minimization run. The response surfaces (Figure 5c–f) indicate a consistent optimal region where high *J_p_* and low F.I. can be achieved simultaneously, supporting agreement between the FCD/RSM and ANN mapping within the investigated domain. Based on this condition, the experimental validation was performed at 75 kPa and 30 °C as a practical compromise setpoint located in the overlapping region of high flux and low fouling identified by the modeling/optimization analyses, rather than at the single-response extrema. Table 5 compares model predictions at this setpoint with the experimental validation results.

The FCD model predicts 611.48 ± 13.93 L h^−1^ m^−2^ for *J_p_* and 81.43 ± 0.43% for F.I, while the ANN provides point predictions of 650.04 L h^−1^ m^−2^ and 80.50%, respectively. The experimental run yielded 605.32 ± 15.34 L h^−1^ m^−2^ and 82.79 ± 1.35%, indicating that both models capture the overall response level in the tested operating window, with some deviations that are expected given the limited experimental domain and the sensitivity of coupled transport–fouling behavior to small shifts in operating conditions. From an engineering standpoint, presenting both RSM/FCD and ANN results strengthens confidence in the identified operating window because the conclusions are supported by two independent modeling strategies that converge on a similar optimal region, which is consistent with recent discussions on hybrid use of mechanistic/statistical and AI-based tools in membrane fouling and process optimization studies [32,69].

### 3.2. Assessment of Permeate Flux and Fouling

**Figure 6:**
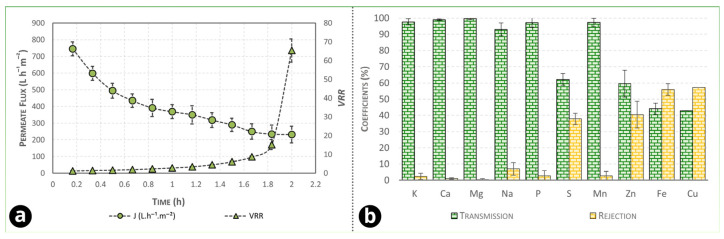
(**a**) Permeate flux evolution during tangential microfiltration of coconut water in concentration mode (*VRR* > 1); (**b**) transmission and rejection coefficients (%) of minerals through the SiC microfiltration membrane.

After obtaining the optimal operating point for green coconut water microfiltration using the SiC multichannel membrane, additional experiments were carried out under the selected conditions to (i) evaluate the evolution of permeate flux and volumetric reduction factor over time under concentration mode (*VRR* > 1) and (ii) assess membrane selectivity in terms of mineral transmission/rejection. The results are shown in Figure 6a,b.

The average permeate flux during concentration was 393.94 L h^−1^ m^−2^ and showed a sharp decline in the initial phase of the operation, followed by a more gradual decrease as the VRR increased (Figure 6a). This behavior is typical of crossflow microfiltration of complex aqueous matrices, in which the early flux drop is associated with rapid establishment of a polarized layer and early pore-interaction phenomena, while the subsequent slower decay is governed by surface-layer growth, compaction, and hydrodynamic stabilization. In this study, the flux-decline behavior was further interpreted using (i) resistance-in-series analysis based on hydraulic permeability (L^P^) at each step of operation and (ii) Hermia-type blocking laws, which are widely used as semi-empirical descriptors of fouling regimes under constant-pressure filtration [53].

According to Table S3 (Supplementary Material), the dominant contribution to the overall hydraulic resistance was concentration polarization (R_C_) (R_C_ > R_F_ > R_M_). This is consistent with the operational observation that most of the flux decline during the run is governed by a reversible surface layer rather than irreversible pore damage. In practice, the high porosity of the SiC structure (≈40%) and its chemical/thermal robustness favor effective cleaning, particularly when combined with elevated cleaning temperatures, and can help restore permeability after operation. A similar resistance ranking (R_C_ > R_F_ > R_M_) was reported by Ghosh et al. [48] for tangential microfiltration of jamun (Indian blackberry) juice. Importantly, the fouling index (FI) values reported here (e.g., ≈91% in-run flux decline) quantify the extent of flux reduction during operation relative to the initial flux and therefore include the effect of reversible phenomena (especially R_C_), which is consistent with the resistance-in-series results indicating R_C_ as the preponderant resistance.

Beyond hydraulic performance, Figure 6b provides an experimental assessment of membrane selectivity regarding mineral passage. As expected for microfiltration (0.2 µm) where dissolved ions are substantially smaller than the nominal pore size, most minerals showed very high transmission (close to quantitative passage) and correspondingly low rejection, indicating that nutritionally relevant electrolytes are largely preserved in the microfiltered product. The few cases with higher apparent rejection (notably for some trace elements) can be explained by their partial association with colloidal/particulate fractions, complexation with macromolecular material, or interaction/adsorption within the polarized layer that behaves as a dynamic secondary membrane. This interpretation is consistent with the predominance of R_C_: once a surface layer develops, it becomes the main hydraulic barrier and can also alter apparent selectivity by retaining species that are not intrinsically size-excluded by the clean membrane pores [48]. In this sense, the same polarized layer that drives flux decline may also contribute to selective retention of mineral-bound colloids, which can be beneficial for clarification while maintaining high transmission of the major dissolved mineral fraction.

To identify the predominant fouling mechanisms during operation, Hermia models were applied to the flux-decline data. Based on the initial filtration phase, there was (as expected) a decline in flux due to concentration polarization [74]. Table S4 (Supplementary Material) reports the estimated constants for the Hermia mechanisms—complete pore blocking (*ε_C_*), intermediate blocking (*ε_I_*), standard blocking (*ε_S_*), and cake layer formation (*ε_CL_*), as well as the corresponding coefficients of determination (*R*^2^). In this framework, *ε_C_* and *ε_S_* are associated with surface/pore-interaction fouling contributions, εI relates to internal/intermediate pore-interaction behavior, and *ε_CL_* is associated with resistance due to cake/gel-layer development [75]. In general, the blocking-related constants (*ε_C_*, *ε_I_*, and *ε_S_*) tend to decrease as the process proceeds, while *ε_CL_* increases with time, consistent with progressive buildup and consolidation of a secondary layer (often described as a gel/polarized layer) at longer filtration times.

**Figure 7:**
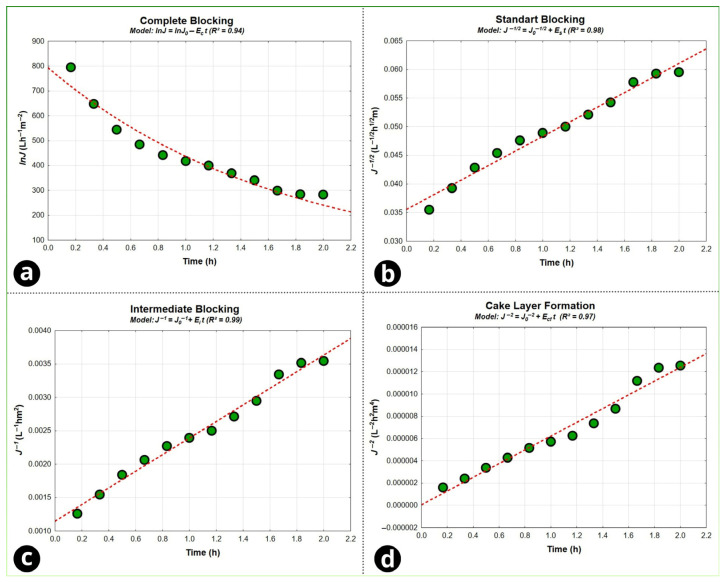
Regression of Hermia models: (**a**) complete blocking, (**b**) standard blocking, (**c**) intermediate blocking, and (**d**) cake layer formation.

Overall, the Hermia regressions (Figure 7 and Table S4) indicate that standard and intermediate blocking behaviors provide the most representative descriptions of the observed flux-decline profile within the evaluated operating time, with cake filtration also contributing as the process advances. The relatively close R^2^ values among the best-fitting mechanisms are compatible with the run duration (2.0 h) and the fact that true “saturation” may not have been fully reached; under such conditions, mixed regimes are expected, where early-stage blocking overlaps with the establishment and gradual compaction of a surface layer. These findings corroborate the resistance analysis in Table S3, where RC (the polarized layer) was the main factor responsible for the overall flux decline. From a mechanistic standpoint, the rapid initial decline followed by a slower decay reflects the transition from early deposit establishment/pore interactions to a regime dominated by growth and compaction of a dynamic surface layer, which behaves as a secondary membrane and increases hydraulic resistance [52]. While this can aid clarification and influence apparent mineral rejection, it also increases energy demand and may raise cleaning frequency requirements [76]. Hermia-type blocking laws remain widely used to differentiate between complete blocking, intermediate blocking, standard blocking, and cake filtration contributions in beverage and juice microfiltration applications [51,77].

For highly porous ceramic structures, including silicon carbide membranes, the literature reports high initial permeabilities and strong chemical/thermal robustness; however, the practical advantage depends on controlling deposit buildup through conservative operating windows, appropriate hydrodynamics, and effective cleaning strategies [19,78]. Finally, the optimization outcomes in Section 3.1 and Section 3.2 have direct implications for the biorefinery-level economics discussed in Section 3.3. At a fixed production target, higher sustainable flux reduces required membrane area and, consequently, the number of modules, pumps, and ancillary equipment, lowering installed costs for the microfiltration step. In parallel, operating conditions that mitigate deposit buildup reduce cleaning frequency, chemical consumption, and downtime, improving effective capacity utilization and decreasing variable OPEX. Therefore, the pressure–temperature window identified here should be interpreted not only as a technical optimum but also as an economic lever influencing both CAPEX sizing and OPEX intensity in the integrated green coconut biorefinery model.

### 3.3. Economic Feasibility

**Figure 8:**
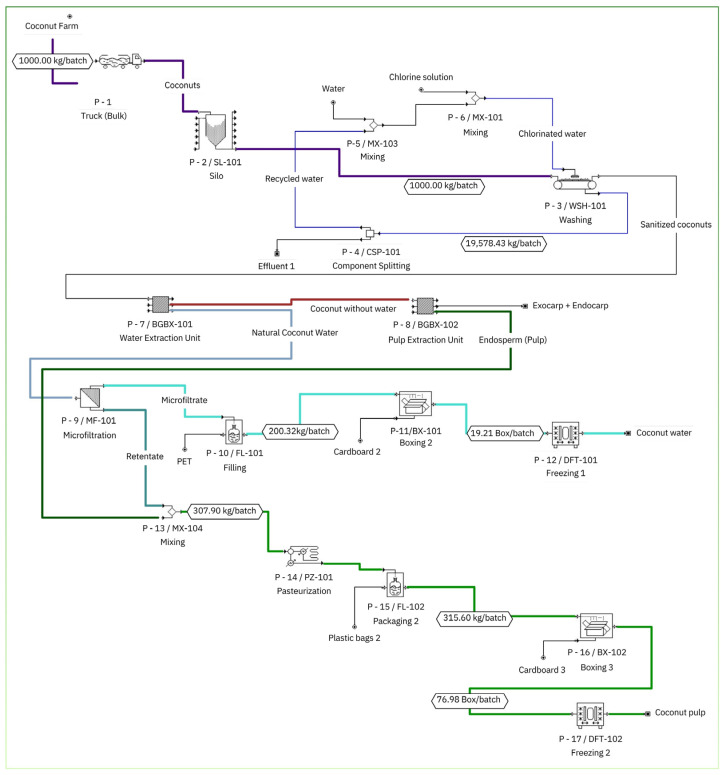
Simulation of the green coconut biorefinery flowchart.

The GCB was modeled in SuperPro Designer Version 11 to integrate process routes for microfiltered coconut water and coconut pulp, to size the main equipment, and to estimate techno-economic indicators that can support implementation decisions. The plant was organized into three main sections (Figure 8): receiving and sanitization (Section 1), raw material processing and phase separation (Section 2), and product processing, packaging, and storage (Section 3). In Section 1, after reception, the coconuts undergo sanitization, and part of the wash water is recovered for reuse, which reduces the net water demand in the pre-treatment block. In Section 2, two steps were represented to obtain coconut water and separate pulp and husks, using coconut composition as the technical basis [60]. Because SPD supports generic unit operation blocks that may not correspond to a single real operation with full physical detail, the flowsheet includes generic boxes to preserve mass balance consistency and sequencing, which is aligned with established approaches for SPD-based process teaching and modeling [79]. In Section 3, coconut water is sent to membrane microfiltration (P-9), reported at a permeate flux of 300 L h^−1^ m^−2^ (concentration mode; *VRR* > 1). This value was adopted as a conservative design flux for equipment sizing under *VRR* > 1, representing a sustained, project-level operating basis that accounts for flux decay during concentration, cleaning/downtime allowances, and operational variability, rather than the best instantaneous or short-run (2 h) flux observed. Therefore, the higher flow values reported in the previous section (Section 3.2) should be interpreted as experimental performance under pilot plant operating conditions, while the economic model uses a conservative yield basis to avoid overestimating capacity.

The permeate is then filled, packed, and stored under refrigeration. The retentate is directed to the pulp stream, producing a combined “pulp” product that is pasteurized, packaged, and refrigerated. The use of membrane filtration as a “cold” stabilization option for coconut water is consistent with studies that evaluate how membrane properties and operating conditions affect performance and product quality [80,81].

Techno-economic assessment is widely used to estimate investment attractiveness and identify cost and risk drivers, especially when a process is still evolving and design choices are being refined [82]. Within this context, the equipment sizing generated by SPD provides an initial engineering and cost basis for the proposed route, consistent with recent work on coconut-related valorization and biorefinery configurations [83]. Table S5 (Supplementary Material) lists the main equipment and unit costs, including the microfilter (MF-101), pasteurizer (PZ-101), fillers, washer, silo, and discrete modules, plus unlisted items, yielding an equipment purchase cost of US$135,000. Importantly, this screening-level TEA focuses on an integrated flowsheet and baseline cost structure; it does not attempt to resolve long-term performance degradation mechanisms or membrane-life uncertainty in a mechanistic way.

Table S6 (Supplementary Material) presents bulk materials and their annual and per-batch demands (kg year^−1^ and kg batch^−1^). It shows an annual coconut demand of 1,346,000 kg year^−1^ and substantial use of cleaning agents and packaging materials (for example, NaOH solution, chlorine solution, PET, plastic, and cardboard), which is typical for food processes that require sanitation and refrigerated distribution.

Operational outputs include utility use and campaign parameters. The model reports annual electricity consumption of 215 kWh, steam of 10 t year^−1^ (10,000 kg year^−1^), pasteurizer cooling water of 2395 t year^−1^ (2,395,000 kg year^−1^), and refrigerant (freon) of 236 t year^−1^ (236,000 kg year^−1^) in refrigerated chambers. The batch time is 18.56 h with 1346 batches per year, producing 2574.21 cases per year of coconut water (10 L per case) and 103,226.25 cases per year of pulp (4 kg per case). To supply this scale, the plant requires 1346 t year^−1^ of green coconuts. To contextualize feedstock availability at the national level, FAOSTAT (UNdata, “Coconuts, in shell”, Production) indicates Brazil is a major producer, and the modeled demand represents a small fraction of national output when compared with the latest year available in that record [4]. Although the physical utility inventories are reported by SPD, the monetary allocation of utilities in the cost breakdown depends on the tariff basis and cost-accounting configuration used in the simulation environment; therefore, the reported utility shares should be interpreted together with the inventory values and the chosen accounting settings.

From an economic perspective, the simulation reports total annual revenue of US$1,588,602, with US$432,468 attributed to microfiltered coconut water and US$1,156,134 to pulp. In the base case, the gross margin is 25.88%, ROI is 33.83%, IRR is 23.80%, NPV is US$733,761, and the payback period is 2.96 years. The use of these metrics as decision criteria is consistent with standard practice in process economics and project evaluation [84,85]. At the same time, the interpretation of IRR benefits from being paired with NPV and the underlying economic assumptions, because IRR has known strengths and limitations depending on cash-flow structure and the decision context [86]. The comparison of payback with a five-year amortization horizon, as cited in the original text in connection with Brazilian financing practice [87], provides an additional practical lens for interpreting return time. It is emphasized that these indicators represent a baseline, screening case assuming steady operation at the adopted design basis; they should not be interpreted as a definitive, risk-complete valuation under all lifetime degradation and maintenance contingencies.

The cost structure supports the interpretation of the performance indicators. Total CAPEX is reported as US$959,000 and annual OPEX as US$1,178,000. Table 6 details the CAPEX composition, with notable contributions from Construction (16.27%), Equipment Purchase Cost (14.08%), Engineering (11.68%), and Working Capital (10.11%), plus contingency, buildings, and other items. This pattern is consistent with early-stage industrial estimates, where indirect and installation-related costs can represent a substantial share of total investment [84,85]. Table 6 details OPEX and shows Raw Materials as the dominant component (56.37%), followed by Labor-dependent (20.80%) and Facility-dependent (13.16%), with additional contributions from Waste treatment/disposal (4.84%) and Transportation (4.67%). This distribution is consistent with processes in which feedstock, packaging, cleaning chemicals, labor productivity, and site overhead are central drivers of unit cost and margins.

In Table 7, Utilities and Consumables each appear as 0.08% of OPEX, while the operational inventory reports meaningful annual use of steam, cooling water, electricity, and refrigerant. This combination can reflect how unit tariffs and accounting allocations were defined in the SPD model, for example, very low unit costs for utilities, allocation of energy and refrigeration costs under Facility-dependent, or specific cost-accounting settings used in the simulation environment. In processes that involve refrigeration, sanitation, and thermal treatment, alignment between physical inventories (consumption) and monetary inventories (costs) is often scrutinized in techno-economic comparisons, and the economic interpretation becomes clearer when the tariff basis and allocation logic used in the model are stated explicitly [84,85].

Price sensitivity was evaluated through scenario analysis (Table 7), which is consistent with sensitivity discussions in bio-based processes [88]. In the base scenario, prices are US$16.80 per case for coconut water and US$11.20 per case for pulp. The +20% scenario increases IRR and NPV and reduces payback, while the −20% scenario decreases IRR, yields a negative NPV, and increases payback. Such behavior is expected because revenues directly scale with selling price, and this effect is often pronounced when raw materials dominate OPEX [89]. The original text also notes that at +20%, the implied retail-level prices approach those observed in commerce, which can influence competitiveness once distribution margins, retail costs, and taxes are considered. In this setting, intermediate scenarios such as +10% allow discussion of economically meaningful improvements while remaining closer to market references used to ground the selling prices.

### 3.4. Technical Challenges and Scale-Up/Integration Considerations

The optimized operating window (pressure–temperature) provides a practical basis for industrial implementation, but scale-up of crossflow microfiltration requires attention to hydrodynamics, cleaning logistics, and long-term performance stability. In existing coconut-water plants, the microfiltration step can be integrated downstream of reception/sanitization and coarse clarification, operating as a cold-stabilization and polishing stage before packaging and refrigerated storage. In this configuration, the main operational targets are (i) sustaining permeate flux at production scale through adequate crossflow velocity and pressure control, and (ii) limiting the buildup of the polarized layer that drives resistance and affects capacity over time. In practice, this means sizing pumps and recirculation loops to maintain shear, implementing tight temperature control, and using monitoring signals (flux trend, TMP drift, and cleaning triggers) to avoid running the system deep into limiting-flux regimes.

From an engineering standpoint, the largest technical risks for continuous operation are long-term flux decay, variability of feed composition between seasons/batches, and the escalation of CIP frequency as deposits become more compact. These effects directly influence effective uptime, cleaning chemical consumption, and utility demand, and therefore should be treated as key scale-up uncertainties. Although silicon carbide membranes are typically higher-cost components, their industrial justification is tied to chemical/thermal robustness and cleaning tolerance; however, a study of membrane lifetime (replacement schedule and degradation curve) was not supported by the current data et and is recognized as a limitation rather than an optimistic assumption.

Accordingly, the present analysis should be interpreted as a preliminary TEA that establishes an internally consistent mass/energy and cost baseline and identifies primary cost drivers. Sensitivity analysis was restricted to the selling price (±10%) as a first-order uncertainty screen. Additional uncertainties, membrane replacement intervals, long-term capacity loss due to flux decline, CIP escalation, downtime, and energy tariff variability remain relevant and should be evaluated in future work when site-specific tariffs, membrane lifetime data, and maintenance records (or validated assumptions) are available. Finally, the current biorefinery scope is limited to coconut water and a combined pulp stream; the valorization of the exocarp and endocarp (e.g., fiber-based materials, bioenergy, or other coproduct routes) was not included and represents a clear opportunity to improve resource efficiency and the economic resilience of the integrated concept.

## 4. Conclusions

The study concludes the following:

**1**  Process optimization and validated operating point:
  - Crossflow microfiltration of green coconut water using a SiC membrane was modeled and optimized using quadratic RSM (FCD) and ANN–GA within the investigated pressure–temperature domain.
  - Both approaches converged to the same optimum, supporting robustness of the selected setpoint: 75 kPa and 30 °C.
  - Experimental validation at this setpoint (*VRR* = 1) produced 605.32 ± 15.34 L m^−2^ h^−1^ permeate flux and 82.79 ± 1.35% fouling index.
**2**  Model performance and what it means for generalization:
  - At the sample/replicate level, ANN achieved higher *R*^2^ and lower fitting errors than FCD, indicating closer within-dataset agreement.
  - Under grouped cross-validation at the condition level, predictive performance favored the quadratic RSM baseline for both responses (*Q^2^* and *RMSE_CV_*), and ANN showed response-dependent generalization, including negative Q^2^ for permeate flux in the CV setting.
  - Take-home message on modeling: ANN is useful as a nonlinear surrogate for within-domain mapping/optimization when supported by replicate data and strict validation, but its predictive advantage should not be generalized beyond the sampled conditions.
**3**  Fouling behavior and mechanistic interpretation:
  - Flux decline followed the expected pattern for complex beverages under crossflow microfiltration.
  - Resistance-in-series analysis indicated concentration polarization as the dominant hydraulic contribution, consistent with the observed loss in permeate flux during operation.
  - Hermia-model fitting indicated partial pore blocking as the predominant semi-empirical descriptor among the tested mechanisms, consistent with deposition/interaction of suspended and colloidal material near the membrane surface.
**4**  Techno-economic feasibility (TEA) and its boundary conditions:
  - The SuperPro Designer^®^ TEA indicates feasibility under baseline assumptions (NPV > 0; IRR = 23.80%; payback = 2.96 years) and shows profitability sensitivity to selling price (±10% remains attractive; larger reductions weaken viability).
  - The economic interpretation should be read as preliminary, because it does not model time-dependent membrane performance degradation, replacement schedules, or site-specific utility tariffs.
**5**  Commercialization challenges, integration, and future work:
  - Scale-up and integration into existing coconut-water facilities will require hydrodynamic control to limit concentration polarization, validated CIP protocols and realistic cleaning frequency, membrane lifetime and replacement planning, and utility-cost realism, especially for refrigeration and thermal steps in the integrated biorefinery.
  - Future work should prioritize condition-wise validation on expanded datasets, inclusion of long-term flux stability and cleaning cycles, and membrane-lifetime–explicit economic scenarios (replacement interval, degradation curve, downtime) to reduce uncertainty in industrial projections.

Overall, the study supports moving forward with implementation and provides clear priorities to reduce risk, cut costs, and improve long-term market performance and resource efficiency.

## Figures and Tables

**Table 1:** Experimental and predicted values in the face-centered design (FCD) methodology and the artificial neural network (ANN) methodology of the experimental design.

Exp.	Independent Variables				Dependent Variables					
	Pressure		Temperature		Permeate Flux			Fouling Index		
	(kPa)		(°C)		(L h^−1^ m^−2^)			(%)		
	*X*_1_	*x*_1_	*X*_2_	*x*_2_	*Y*_1_			*Y*_2_		
					Experimental	RSM	ANN	Experimental	RSM	ANN
01	50	−1.0	20	−1.0	324.35 ± 06.97	347.53 ± 25.96	324.29 ± 0.00	84.55 ± 1.35	83.97 ± 0.77	84.58 ± 0.00
02	200	+1.0	20	−1.0	302.26 ± 09.45	309.02 ± 25.96	302.56 ± 0.00	93.28 ± 0.86	94.09 ± 0.77	93.56 ± 0.00
03	50	−1.0	40	+1.0	471.78 ± 11.53	468.75 ± 25.96	470.60 ± 0.00	82.92 ± 1.04	82.46 ± 0.77	82.74 ± 0.00
04	200	+1.0	40	+1.0	527.11 ± 14.07	507.45 ± 25.96	527.65 ± 0.00	94.66 ± 1.21	95.59 ± 0.77	94.05 ± 0.00
05	50	−1.0	30	0.0	608.78 ± 16.77	550.10 ± 18.30	638.28 ± 0.00	78.21 ± 0.67	79.50 ± 0.48	78.18 ± 0.00
06	200	+1.0	30	0.0	498.88 ± 03.98	550.10 ± 18.30	498.32 ± 0.00	92.61 ± 0.66	91.12 ± 0.48	92.50 ± 0.00
07	125	0.0	20	−1.0	469.00 ± 12.34	438.86 ± 20.01	469.41 ± 0.00	88.98 ± 1.08	89.03 ± 0.61	88.61 ± 0.00
08	125	0.0	40	+1.0	575.90 ± 03.09	598.59 ± 20.01	575.88 ± 0.00	89.78 ± 0.80	89.03 ± 0.61	89.54 ± 0.00
09 (c)	125	0.0	30	0.0	656.47	660.59 ± 12.63	659.19 ± 0.00	84.10	85.31 ± 0.38	84.76 ± 0.00
10 (c)	125	0.0	30	0.0	678.96			86.42		
11 (c)	125	0.0	30	0.0	639.43			84.06		
12 (c)	125	0.0	30	0.0	645.50			85.56		
13 (c)	125	0.0	30	0.0	660.21			85.80		

(x) represents the coded level of variables, (X) represents the actual level of variables, and (c) = central point. Single homogenized batch; 9 unique conditions (8 + center point). Non-center points in triplicate (*n* = 3) and center point in quintuplicate (*n* = 5; samples 9–13), totaling 29 runs; values are mean ± SD.

**Table 2:** Mathematical models of hydraulic resistances and permeance coefficients of microfiltration processes.

Fouling Mechanisms		Final Equation		Unit
$L_{P}^{0}$	Hydraulic permeance coefficient of a clean membrane	LP0=Jw0∆PTM	(8)	mPa^−1^ s^−1^
$L_{P}^{1}$	Coefficient of hydraulic permeance after the process	LP1=Jw1∆PTM	(9)	mPa^−1^ s^−1^
$L_{P}^{2}$	Coefficient of hydraulic permeance after physical cleaning	LP2=Jw2∆PTM	(10)	mPa^−1^ s^−1^
$L_{P}^{3}$	Coefficient of hydraulic permeance after chemical cleaning	LP3=Jw3∆PTM	(11)	mPa^−1^ s^−1^
$R_{T}$	Total system resistance	$R_{T}=R_{M}+R_{C}+R_{F}$	(12)	m^−1^
$R_{M}$	Membrane resistance	RM=1µwLp0	(13)	m^−1^
$R_{C}$	Concentration polarization resistance	RC=1µw(1Lp1−1Lp2)	(14)	m^−1^
$R_{R}$	Reversible resistance	RR=1µw(1Lp2−1Lp3)	(15)	m^−1^
$R_{I}$	Irreversible resistance	RI=1µw(1Lp3−1Lp0)	(16)	m^−1^
$R_{F}$	Fouling resistance	$R_{F}=R_{R}+R_{I\;}$	(17)	m^−1^
F.I	Fouling index	F.I=1−Lp1Lp0×100	(18)	%

“µ_w_” is the viscosity of water (1.0 mPa s), and “ΔPTM” is the transmembrane pressure of the system (kPa) [48,49,50].

**Table 3:** Overview of the fouling mechanisms of microfiltration processes.

Fouling Mechanisms	*n*	Model		Constant (ε)		Representation
Complete blockage of pores	2.0	$lnJ={lnJ}_{0}-\varepsilon_{C}t$	(19)	$\varepsilon_{C}=K_{C}v_{0}$	(23)	** 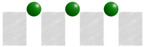 **
Standard pore blockage	1.5	$J^{-1/2}=J_{0}^{-1/2}+\varepsilon_{P}$	(20)	εS=KPv0J0	(24)	** 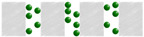 **
Intermediate blockage of pores	1.0	$J^{-1}=J_{0}^{-1}+\varepsilon_{i}t$	(21)	εI=KCv0J01/2	(25)	** 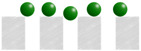 **
Cake layer formation	0.0	$J^{-2}=J_{0}^{-2}+\varepsilon_{CG}t$	(22)	εCL=(2RR)KCLv0J02	(26)	** 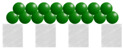 **

“*J*” is the permeate flux (L h^−1^ m^−2^); “*J*_0_” is the initial permeate flux (L h^−1^ m^−2^); “*t*” is the time (h); “*R_R_*” is the ratio of cake resistance over clean membrane resistance (dimensionless); “*v*_0_” is the initial velocity per unit surface area per membrane surface (m h^−1^); “*K_C_*” is the surface area blocked by the membrane per total number of permeates in the membrane unit (m^−1^); “*K_P_*” is the reduction in pore cross-sectional area per total unit of permeate flux (m^−1^); “*K_CL_*” is the area of cake formed per unit volume of permeate (m^−1^); “*ε_C_*” is the constant of complete blockage (s^−1^); “*ε_P_*” is the standard blocking constant (s^−1/2^ m^−1/2^); “*ε_I_*” is the intermediate pore blockage constant (m^−1^); and “*ε_CL_*” is the cake formation constant (s m^−2^) [54,55].

**Table 4:** Predicted and experimental results of the experimental design in the studied methodologies (FCD–ANN).

**Face Centered Design—FCD**									
Dependent Variables	*AAD*	*MSE*	*RMSE*	*MPE*	*NMSE*	*NRMSE*	*R* ^2^	*Q* ^2^	*RMSE_CV_*
Permeate Flux	23.81	856.01	29.26	4.65	1.58	0.05	0.93	0.37	94.34
Fouling Index	0.72	0.71	0.84	0.83	0.01	0.01	0.95	0.69	2.84
**Artificial Neural Network—ANN**									
Dependent Variables	*AAD*	*MSE*	*RMSE*	*MPE*	*NMSE*	*NRMSE*	*R* ^2^	*Q* ^2^	*RMSE_CV_*
Permeate Flux	7.80	101.01	10.05	1.58	0.20	0.01	0.99	−0.59	146.93
Fouling Index	0.79	1.03	1.01	0.90	0.01	0.01	0.96	0.34	4.02

Sample-level metrics (*AAD*, *MSE*, *RMSE*, *MPE*, *NMSE*, *NRMSE*, *R*^2^): calculated using all individual observations (replicate-level data; *n* = 29 samples), comparing experimental values (X_A_) and model predictions (X_P_) for each sample. Here, X_M_ is the mean of the experimental samples for the response, and *n* is the number of samples. Condition-wise cross-validation (*Q*^2^ and *RMSE_CV_*): calculated using leave-one-condition-out grouped validation across the 13 unique pressure–temperature conditions, where each fold holds out all replicates of one condition (no replicate leakage). Predictions from all held-out folds are pooled to compute *Q*^2^ and *RMSE_CV_* against the corresponding experimental samples.

**Table 5:** Values of all statistical parameters of the experimental design in the studied methodologies (FCD–ANN).

Dependent Variables	Prediction (FCD)	Prediction (ANN) ^‡^	Experimental Data
Permeate Flux (L h^−1^ m^−2^)	611.48 ± 13.93	650.04	605.32 ± 15.34
Fouling Index (%)	81.43 ± 0.43	80.50	82.79 ± 1.35

Process Conditions: 75 kPa and 30 °C; ^‡^ ANN values are point predictions; predictive uncertainty is discussed using cross-validated error metrics (e.g., *RMSE_CV_*) and sample-level residual metrics.

**Table 6:** Composition of CAPEX and OPEX.

**Capex**		
**Component**	**$**	**%**
Equipment Purchase Cost	135,000	14.08
Installation	57,000	5.94
Process Piping	47,000	4.90
Instrumentation	54,000	5.63
Insulation	4000	0.42
Electrical	14,000	1.46
Buildings	61,000	6.36
Yard Improvement	20,000	2.09
Auxiliary Facilities	54,000	5.63
Engineering	112,000	11.68
Construction	156,000	16.27
Contractor’s Fee	36,000	3.75
Contingency	71,000	7.40
Working Capital	97,000	10.11
Startup Cost	41,000	4.28
Total	959,000	100.00
**Opex**		
**Component**	**$**	**%**
Raw Materials	664,000	56.37
Labor-Dependent	245,000	20.80
Facility-Dependent	155,000	13.16
Consumables	1000	0.08
Waste Treatment/Disposal	57,000	4.84
Utilities	1000	0.08
Transportation	55,000	4.67
Total	1178.000	100.00

**Table 7:** Sensitivity analysis.

Scenario	Relative Price Variation vs. Base	Coconut Water	Pulp	IRR	NPV	Payback
	(%)	(US$/Case)	(US$/Case)	(%)	(US$)	(Years)
Base case	0	16.80	11.20	23.80	733,761	2.96
Scenario 1	+20	20.16	13.44	36.87	1,779,838	1.86
Scenario 2	+10	18.48	12.32	30.58	1,256,800	2.29
Scenario 3	−10	15.12	10.08	16.36	210,723	4.18
Scenario 4	−20	13.44	8.96	7.78	−286,402	7.15

## Data Availability

The original contributions presented in this study are included in the article/Supplementary Materials. Further inquiries can be directed to the first author (diogo.rocha@posgrad.ufsc.br).

## Supplementary Materials

The following supporting information can be downloaded at: https://www.mdpi.com/article/10.3390/foods15040623/s1, Figure S1: Design points of a two-factor face-centered design: factorial, axial, and center runs; Figure S2: Silicon carbide tangential microfiltration system; Figure S3: Histogram of errors in the response (a) permeate flux and (b) fouling index, of the neural network post-training, and coefficients of determination (*R*^2^) of the training (c), validation (d), test (e), and all (f) data from the responses (outputs) of the ANN; Table S1: Regression coefficients of the dependent variables of the experimental design before and after reparameterization; Table S2: ANOVA of the dependent variables of the experimental design; Table S3: Determination of hydraulic permeances (L^0^_P_-L^3^_P_), resistances (R_T_, R_M_, R_C_, R_R_ and R_I_), and fouling index (F.I) of the coconut water microfiltration process in concentration mode; Table S4: Values of the parameters fitted by the Hermia model and the respective coefficients of determination (*R*^2^) related to fouling; Table S5: Equipment; Table S6: Bulk materials.

## Abbreviations

The following abbreviations are used in this manuscript:

AAD	Average absolute deviation
A_M_	Membrane area (m^2^)
ANN	Artificial neural network
ANN–GA	Artificial neural network–genetic algorithm
ANOVA	Analysis of variance
CA	Feed concentration
CAPES	Coordenação de Aperfeiçoamento de Pessoal de Nível Superior (Brazil)
CAPEX	Capital expenditure
CBI	Centre for the Promotion of Imports
CFMF	Crossflow microfiltration
CIP	Clean in place
CP	Permeate concentration
CV	Cross-validation
C_6_H_8_O_7_	Citric acid
C_0_	Cost of the reference equipment
EPC	Equipment purchase cost
FAO	Food and Agriculture Organization of the United Nations
FCD	Face-Centered Design
F.I.	Fouling index
GA	Genetic algorithm
GCB	Green coconut biorefinery
HNO_3_	Nitric acid
IRR	Internal rate of return
J_0_	Initial permeate flux
J_p_	Permeate flux
K_2_S_2_O_5_	Potassium metabisulfite
K_C_	Complete blocking model constant
K_CL_	Cake layer model constant
K_P_	Standard blocking model constant
L^0^_P_	Hydraulic permeance (clean membrane)
L^1^_P_	Hydraulic permeance (after process)
L^2^_P_	Hydraulic permeance (after physical cleaning)
L^3^_P_	Hydraulic permeance (after chemical cleaning)
MAD	Mean absolute deviation
MFF	Multilayer feed-forward (neural network)
MPE	Mean percentage error
MRR	Minimum rate of return
MSE	Mean squared error
MTE	Ministry of Labor and Employment (Brazil)
NaClO	Sodium hypochlorite
NaOH	Sodium hydroxide
NMSE	Normalized mean squared error
NPV	Net present value
NRMSE	Normalized root mean squared error
OPEX	Operating expenditure
PET	Polyethylene terephthalate
Q	Desired capacity
Q^2^	Cross-validated predictive coefficient of determination.
*R*^2^	Coefficient of determination
R_C_	Concentration-polarization resistance
R_F_	Fouling resistance
R_I_	Irreversible resistance
R_M_	Intrinsic membrane resistance
RMSE	Root mean squared error
RMSE_CV_	Cross-validated root mean squared error.
ROI	Return on investment
R_R_	Reversible resistance
RSM	Response surface methodology
R_T_	Total resistance
SELIC	Sistema Especial de Liquidação e Custódia (Brazilian reference interest rate)
SiC	Silicon carbide
SPD	SuperPro Designer^®^
t	Time (h)
tansig	Hyperbolic tangent sigmoid transfer function
trainlm	Levenberg–Marquardt backpropagation training function
purelin	Linear transfer function
mapminmax	min–max normalization function
US$	United States dollar
v_0_	Initial superficial velocity (per membrane area)
V_F_	Total feed volume (L)
V_P_	Permeate volume collected (L)
V_R_	Retentate volume (L)
VRR	Volume Reduction Ratio
ΔP_TM_	Transmembrane pressure
µ_w_	Water viscosity
ε_C_	Complete blocking kinetic constant
ε_CL_	Cake layer kinetic constant
ε_I_	Intermediate blocking kinetic constant
ε_P_	Standard blocking kinetic constant
