# Supplementary material for: Green Coconut Biorefinery: RSM and ANN–GA Optimization of Coconut Water Microfiltration with IntegratedTechno-Economic Analysis
Source: Foods. 2026 Feb 9;15(4):623. doi: 10.3390/foods15040623 (PMC12939026; doi:10.3390/foods15040623)
Supplement: Supplementary file 1 [file foods-15-00623-s001.zip › foods-4103850-supplementary.pdf]

foods

Article

Green Coconut Biorefinery: RSM and ANN–GA Optimization of Coconut Water Microfiltration with IntegratedTechno-Economic Analysis

José Diogo da Rocha Viana [1], Moacir Jean Rodrigues [2], Arthur Claudio Rodrigues de Souza [3], Raimundo Marcelino da Silva Neto [3], Paulo Riceli Vasconcelos Ribeiro [3], José Carlos Cunha Petrus [1,*] and Ana Paula Dionísio [3,*]

[1] Programa de Pós-Graduação em Engenharia de Alimentos, Universidade Federal de Santa Catarina—UFSC, Florianópoli 88040-900, Brazil; diogo.rocha@posgrad.ufsc.br
[2] Programa de Pós-Graduação em Ciências Naturais, Universidade Estadual do Ceará—UECE, Fortaleza 60060-120, Brazil
[3] Embrapa Agroindústria Tropical—EMBRAPA, Fortaleza 60511-110, Brazil
* Correspondence: jose.petrus@ufsc.br (J.C.C.P.); ana.dionisio@embrapa.br (A.P.D.)

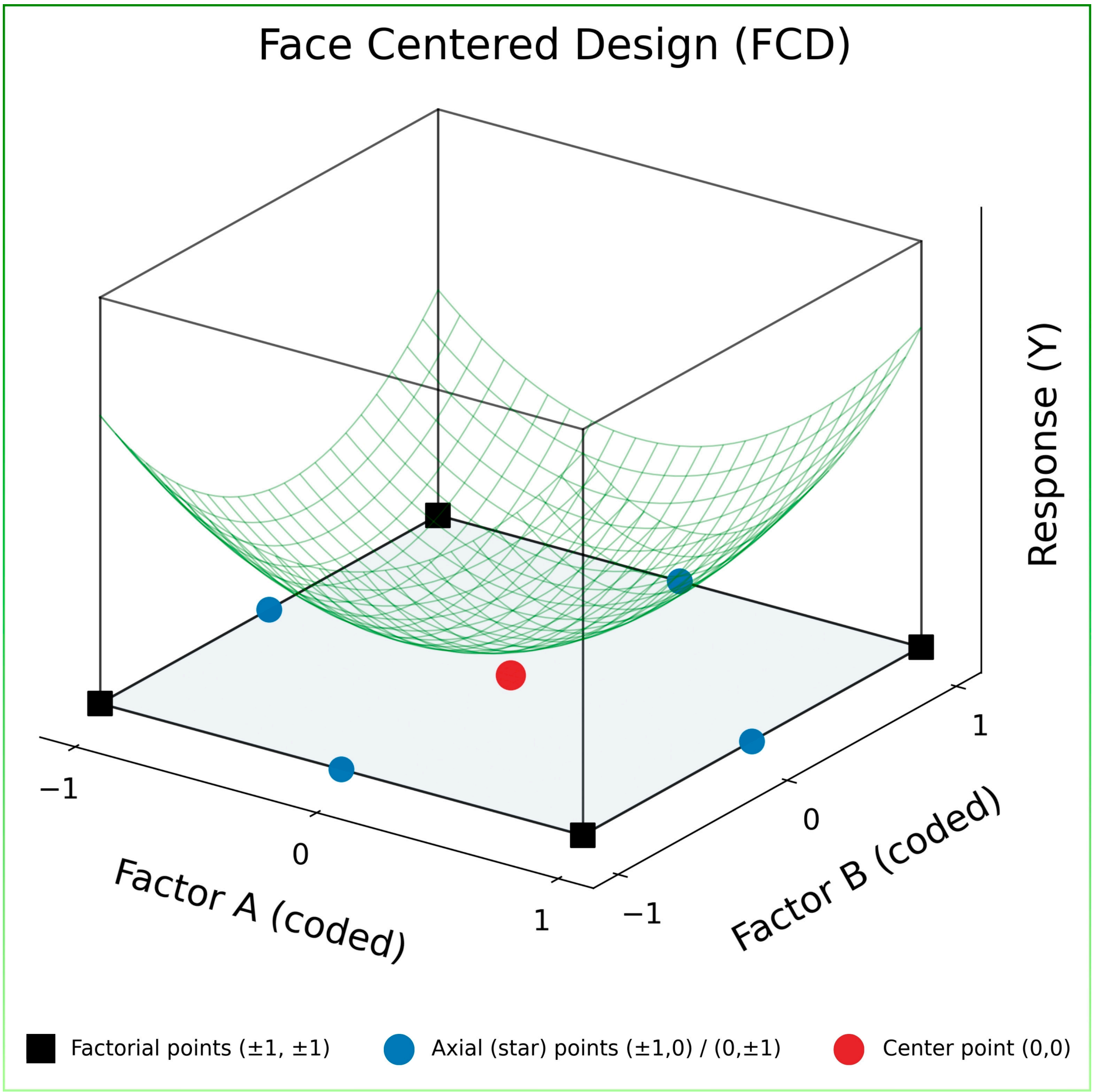

Figure S1. Design points of a two-factor face-centered design: factorial, axial, and center runs.

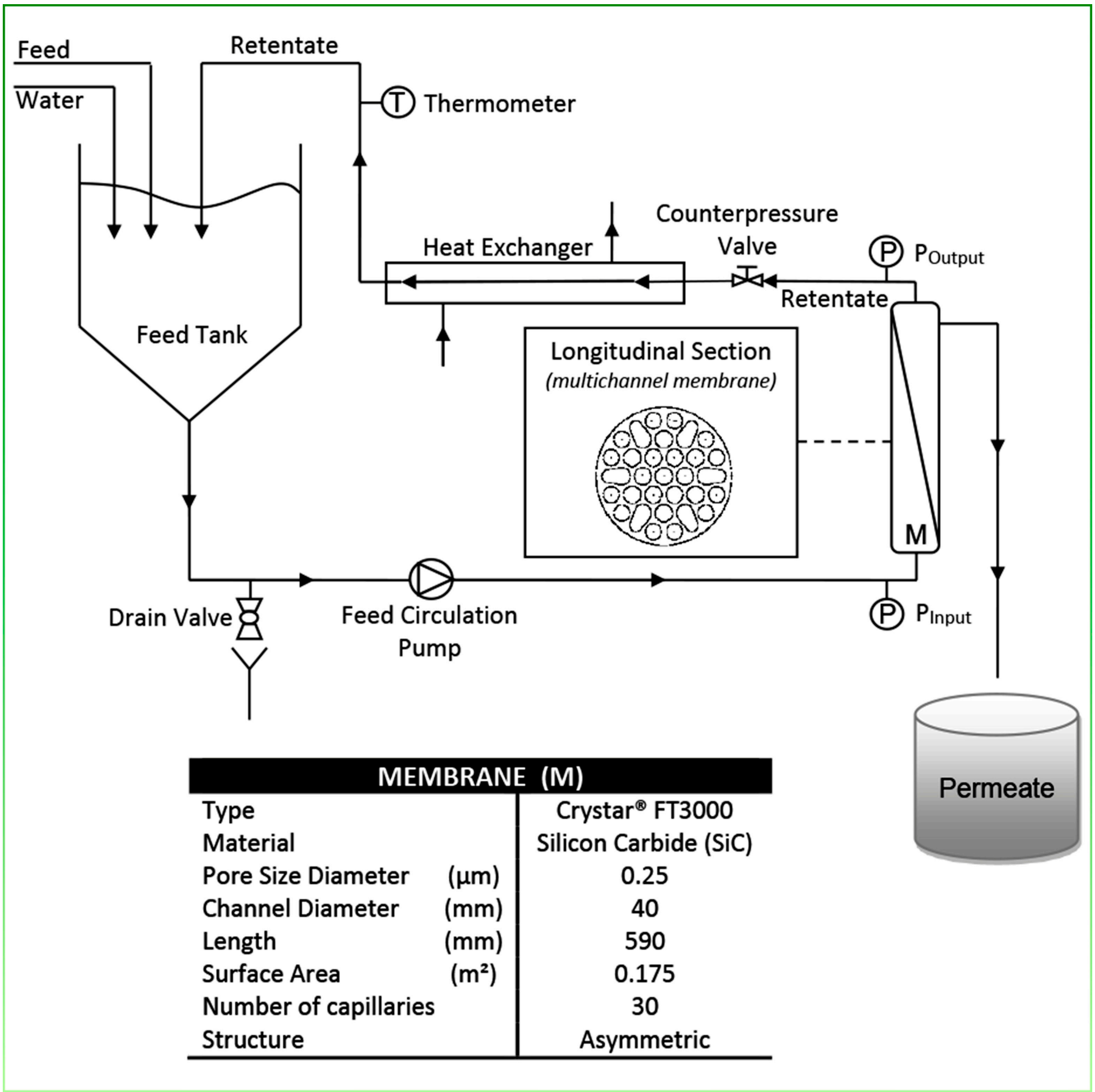

MEMBRANE (M)		
Type		Crystar® FT3000
Material		Silicon Carbide (SiC)
Pore Size Diameter	(µm)	0.25
Channel Diameter	(mm)	40
Length	(mm)	590
Surface Area	(m^2)	0.175
Number of capillaries		30
Structure		Asymmetric

Figure S2. Silicon carbide tangential microfiltration system.

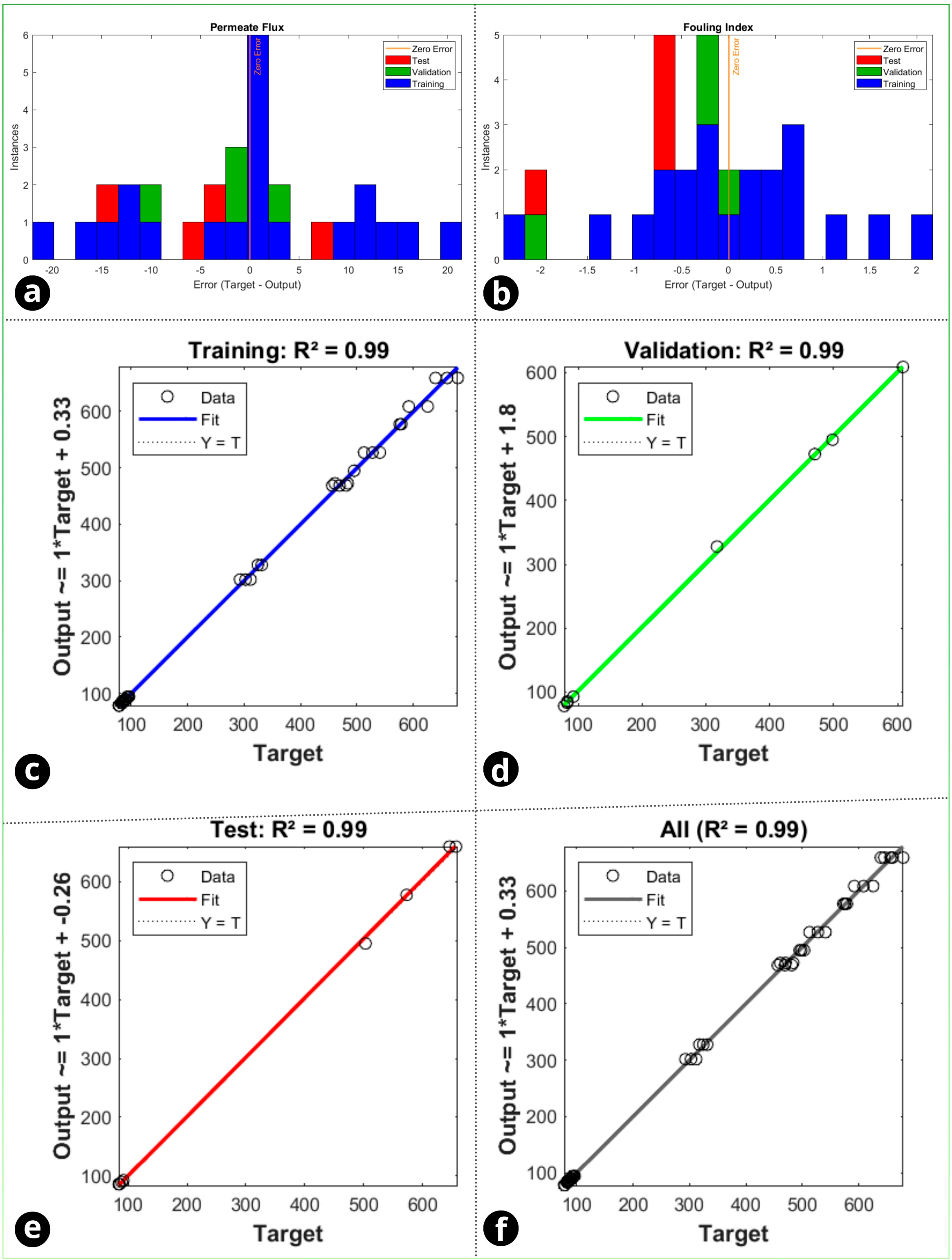

Figure S3. Histogram of errors in the response (**a**) permeate flux and (**b**) fouling index, of the neural network post-training, and coefficients of determination (R^2) of the training (**c**), validation (**d**), test (**e**), and all (**f**) data from the responses (outputs) of the ANN.

Table S1. Regression coefficients of the dependent variables of the experimental design before and after reparameterization.

Permeate Flux (Y_1, L h^{-1} m^{-2})					Fouling Index (Y_2, %)				
**Factors**	**Regression Coefficient**	**Standard Error**	t_{cal}	*p - valor*	**Factors**	**Regression Coefficient**	**Standard Error**	t_{cal}	*p - valor*
Average	660.59	12.21	54.10	0.00	Average	85.43	0.48	178.21	0.00
x_1 (L)	-12.78	7.83	-1.63	0.11	x_1 (L)	5.81	0.31	18.91	0.00
x_1^2 (Q)	-110.48	12.81	-8.63	0.00	x_1^2 (Q)	-0.23	0.50	-0.45	0.65
x_2 (L)	79.86	7.83	10.20	0.00	x_2 (L)	0.09	0.31	0.29	0.77
x_2^2 (Q)	-141.86	12.81	-11.08	0.00	x_2^2 (Q)	3.75	0.50	7.45	0.00
$x_1.x_2$	19.35	9.59	2.02	0.05	$x_1.x_2$	0.75	0.38	2.00	0.05
**Significant Coefficients (Reparametrized)**					**Significant Coefficients (Reparametrized)**				
**Factors**	**Regression Coefficient**	**Standard Error**	t_{cal}	*p - valor*	**Factors**	**Regression Coefficient**	**Standard Error**	t_{cal}	*p - valor*
Average	660.59	12.63	52.31	0.00	Average	85.31	0.38	224.79	0.00
x_1^2 (Q)	-110.48	13.24	-8.34	0.00	x_1 (L)	5.81	0.30	19.59	0.00
x_2 (L)	79.86	8.10	9.86	0.00	x_2^2 (Q)	3.72	0.48	7.72	0.00
x_2^2 (Q)	-141.86	13.24	-10.71	0.00	$x_1.x_2$	0.75	0.36	2.07	0.05
$x_1.x_2$	19.35	9.92	1.95	0.06					

(L) = linear term; (Q) = quadratic term.

Table S2. ANOVA of the dependent variables of the experimental design.

DEPENDENT VARIABLE (Permeate Flux)						
**Source of variation**	**Sum of squares**	**Degrees of freedom**	**Mean square**	F_{test}	*p - value*	R^2
**Regression**	365991.5	4.0	91497.9	77.5	0.00	
**Residuals**	28318.0	24.0	1179.9			
**Lack of fit**	25533.5	4.0	6383.4	45.8	0.00	0.93
**Pure error**	2785.5	20.0	139.2			
**Total**	394309.5	28.0				
**Exp. Model**	$Y_1 = 660.59 - 110.48x_1^2 + 79.86x_2 - 141.86x_2^2 + 19.35x_1x_2$					(01)

DEPENDENT VARIABLE (Fouling Index)						
**Source of variation**	**Sum of squares**	**Degrees of freedom**	**Mean square**	F_{test}	*p - value*	R^2
**Regression**	709.2	3.0	236.4	149.2	0.00	
**Residuals**	39.6	25.0	1.6			
**Lack of fit**	19.6	5.0	3.9	3.9	0.01	0.95
**Pure error**	20.0	20.0	1.0			
**Total**	748.8	28.0				
**Exp. Model**	$Y_2 = 85.31 + 5.81x_1 + 3.72x_2^2 + 0.75x_1x_2$					(02)

$F_{tabuleted\ 4.24} = 2.78$; $F_{tabuleted\ 3.25} = 2.99$;

Y_1 and Y_2 are permeate flux (L h^{-1} m^{-2}) and fouling index (%), respectively, and x_1 and x_2 refer to pressure (kPa) and temperature (°C), respectively.

Table S3. Determination of hydraulic permeances (L^0_P - L^3_P), resistances (R_T, R_M, R_C, R_R and R_I), and fouling index (F.I) of the coconut water microfiltration process in concentration mode.

L^0_P (10^{-8})	L^1_P (10^{-8})	L^2_P (10^{-8})	L^3_P (10^{-8})
(mPa^{-1} s^{-1})			
2,16	0,18	0,34	1,98

R_T (10^{12})	R_M (10^{12})	R_C (10^{12})	R_R (10^{12})	R_I (10^{12})	R_M / R_T	R_C / R_T	R_F / R_T	F.I
(m^{-1})					(%)			
5,59	0,46	2,68	2,41	0,04	8,29	47,88	43,83	91,70

Table S4. Values of the parameters fitted by the Hermia model and the respective coefficients of determination (R^2) related to fouling.

Fouling Mechanism	n	J_0	$\mathcal{E}_C; \mathcal{E}_I; \mathcal{E}_S; \mathcal{E}_{CL}$	R^2
Complete Pore Blocking	2.0	793.70 ± 25.01	0.60 ± 0.03	0.94 ± 0.01^{a}
Standard Blocking	1.5	0.00 ± 0.01	0.00 ± 0.01	0.99 ± 0.02^{b}
Intermediate Blocking	1.0	0.03 ± 0.01	0.01 ± 0.02	0.98 ± 0.02^{b}
Cake Layer Formation	0.0	0.00 ± 0.01	0.00 ± 0.01	0.97 ± 0.01^{ab}

Process Conditions: 75.0 kPa and 30.0 ºC;

n = fouling mechanism constant; J_0 = initial permeate flux (L h^{-1} m^{-2}); R^2 = correlation coefficient; $\mathcal{E}_C$: constant of complete blocking; $\mathcal{E}_S$ = standard blocking constant ($s^{-1/2}$ $m^{-1/2}$); $\mathcal{E}_I$ = constant of the intermediate blocking (m^{-1}); $\mathcal{E}_{CL}$ = constant of the cake layer formation (s m^{-2}). Means followed by the same letters (columns) are not significantly different according to Tukey's test (α = 0.05). Means of three process repetitions.

Table S5. Equipment.

Quantity	Name	Description	Unit Cost. ($)
**1**	MF-101	Microfilter (Membrane Area = 0,32 m^2)	30,000
**1**	PZ-101	Pasteurizer (Rated Throughput = 77,38 L h^{-1})	15,000
**1**	FL-101	Filler (Discrete Throughput = 192,12 entities h^{-1})	10,000
**1**	WSH-101	Washer (Rated Throughput = 2000 kg h^{-1})	10,000
**1**	SL-101	Silo (Vessel Volume = 1309,21 L)	10,000
**1**	FL-102	Filler (Discrete Throughput = 769,75 entities h^{-1})	10,000
**1**	DFT-102	Discrete Module (Vessel Volume = 387,97 L)	7,000
**1**	DFT-101	Discrete Freeze-Thaw Module (Vessel Volume = 240,54 L)	7,000
**1**	BGBX-102	Batch Generic Box (Vessel Volume = 899,09 L)	3,000
**1**	BGBX-101	Batch Generic Box (Vessel Volume = 1116,91 L)	2,000
**1**	BX-102	Packer (Discrete Throughput = 76,98 entities h^{-1})	2,000
**1**	BX-101	Packer (Discrete Throughput = 19,21 entities h^{-1})	2,000
**UNLISTED EQUIPMENT**			27,000
**TOTAL**			135,000

Table S6. Bulk materials.

Material	kg/yr	kg/batch
Coconut	1,346,000	1,000.00
PET	11,171	8.300
Nitrogen	2,868	2.131
Cardboard	18,700	13.893
Plastic	10,361	7.698
Chlorine Solution	134,600	100.000
NaOH Solution	740,300	550.000
Water	9,483,020	7,045.334
**TOTAL**	11,747,019	8,727.355
